# Geospatial disparities in survival of patients with breast cancer in sub-Saharan Africa from the African Breast Cancer-Disparities in Outcomes cohort (ABC-DO): a prospective cohort study

**DOI:** 10.1016/S2214-109X(24)00138-4

**Published:** 2024-05-21

**Authors:** Joanne Kim, Peter M Macharia, Valerie McCormack, Milena Foerster, Moses Galukande, Maureen Joffe, Herbert Cubasch, Annelle Zietsman, Angelica Anele, Shadrach Offiah, Groesbeck Parham, Leeya F Pinder, Benjamin O Anderson, Joachim Schüz, Isabel dos Santos-Silva, Kayo Togawa

**Affiliations:** a Environment and Lifestyle Epidemiology Branch, International Agency for Research on Cancer, WHO, Lyon, France; b Department of Public Health, Institute of Tropical Medicine, Antwerp, Belgium; c Population and Health Impact Surveillance Group, Kenya Medical Research Institute-Wellcome Trust Research Programme, Nairobi, Kenya; d Centre for Health Informatics, Computing and Statistics, Lancaster Medical School, Lancaster University, Lancaster, UK; e College of Health Sciences, Makerere University, Kampala, Uganda; f Strengthening Oncology Services Research Unit, Faculty of Health Sciences, University of the Witwatersrand, Johannesburg, South Africa; g Department of Surgery, University of the Witwatersrand, Johannesburg, South Africa; h AB May Cancer Centre, Windhoek Central Hospital, Windhoek, Namibia; i Federal Medical Centre, Owerri, Nigeria; j Abia State University Teaching Hospital, Aba, Nigeria; k Department of Obstetrics and Gynecology, School of Medicine, University of North Carolina, Chapel Hill, NC, USA; l Department of Obstetrics and Gynecology, University of Washington, Seattle, WA, USA; m Department of Surgery, University of Washington, Seattle, WA, USA; n Department of Non-Communicable Diseases Epidemiology, London School of Hygiene & Tropical Medicine, London, UK; o Division of Surveillance and Policy Evaluation, National Cancer Center Institute for Cancer Control, Tokyo, Japan

## Abstract

**Background:**

There is an urgent need to improve breast cancer survival in sub-Saharan Africa. Geospatial barriers delay diagnosis and treatment, but their effect on survival in these settings is not well understood. We examined geospatial disparities in 4-year survival in the African Breast Cancer-Disparities in Outcomes cohort.

**Methods:**

In this prospective cohort study, women (aged ≥18 years) newly diagnosed with breast cancer were recruited from eight hospitals in Namibia, Nigeria, South Africa, Uganda, and Zambia. They reported sociodemographic information in interviewer-administered questionnaires, and their clinical and treatment data were collected from medical records. Vital status was ascertained by contacting participants or their next of kin every 3 months. The primary outcome was all-cause mortality in relation to rural versus urban residence, straight-line distance, and modelled travel time to hospital, analysed using restricted mean survival time, Cox proportional hazards, and flexible parametric survival models.

**Findings:**

2228 women with breast cancer were recruited between Sept 8, 2014, and Dec 31, 2017. 127 were excluded from analysis (58 had potentially recurrent cancer, had previously received treatment, or had no follow-up; 14 from minority ethnic groups with small sample sizes; and 55 with missing geocoded home addresses). Among the 2101 women included in analysis, 928 (44%) lived in a rural area. 1042 patients had died within 4 years of diagnosis; 4-year survival was 39% (95% CI 36–42) in women in rural areas versus 49% (46–52) in urban areas (unadjusted hazard ratio [HR] 1·24 [95% CI 1·09–1·40]). Among the 734 women living more than 1 h from the hospital, the crude 4-year survival was 37% (95% CI 32–42) in women in rural areas versus 54% (46–62) in women in urban areas (HR 1·35 [95% CI 1·07–1·71] after adjustment for age, stage, and treatment status). Among women in rural areas, mortality rates increased with distance (adjusted HR per 50 km 1·04, 1·01–1·07) and travel time (adjusted HR per h 1·06, 1·02–1·10). Among women with early-stage breast cancer receiving treatment, women in rural areas had a strong survival disadvantage (overall HR 1·54, 1·14–2·07 adjusted for age and stage; >1 h distance adjusted HR 2·14, 1·21–3·78).

**Interpretation:**

Geospatial barriers reduce survival of patients with breast cancer in sub-Saharan Africa. Specific attention is needed to support patients with early-stage breast cancer living in rural areas far from cancer treatment facilities.

**Funding:**

US National Institutes of Health (National Cancer Institute), Susan G Komen for the Cure, and the International Agency for Research on Cancer.

## Introduction

Breast cancer is the most common cancer in women worldwide and is the most frequent cause of cancer death. The rate of 5-year survival can reach 90% in high-income settings compared with 33–70% in sub-Saharan Africa.^1^ Poor breast cancer survival in sub-Saharan Africa can be attributed to several factors, including delays in diagnosis leading to advanced cancers, and limited access to high-quality care.^2^ Although these factors are largely affected by a patient's personal financial resources and broader sociocultural and health-system factors, inequalities in cancer survival might be exacerbated by geospatial barriers such as rural residence and distance from or travel time to a hospital.

In sub-Saharan Africa, the few hospitals with specialised cancer treatment facilities are found in large urban centres and serve a large geographical area. Individuals living in remote areas could face additional barriers to accessing diagnostic and treatment facilities and adhering to cancer treatment plans that require several visits over an extended period. Delays in diagnosis and the initiation and completion of treatment could affect breast cancer survival in these settings. However, data on geospatial disparities in survival within sub-Saharan Africa are sparse. The use of geographical information systems (GIS) in combination with epidemiological, clinical, and treatment data can help to identify geographical disparities in cancer outcomes.^3^ In the African Breast Cancer-Disparities in Outcomes (ABC-DO) cohort, patients with breast cancer from five sub-Saharan African countries—Namibia, Nigeria, South Africa, Uganda, and Zambia—were actively followed up every 3 months. Previous findings from this cohort were that living further from the hospital was associated with being at a later stage of breast cancer at diagnosis,^4^ consistent with individual results from South Africa and Nigeria.^5^, ^6^, ^7^, ^8^ However, to our knowledge, only two studies have examined geospatial characteristics in relation to breast cancer survival in sub-Saharan Africa, finding a higher rate of death associated with rural residence and travel time among patients with breast cancer in Nigeria (n=609) and Ethiopia (n=302).^8^, ^9^ Both constructed their patient cohorts retrospectively, with potentially differential misclassification in the date of censoring or death expected.

**Research in context**

**Evidence before this study**

We searched PubMed using the search terms ((((((geospatial) OR (rural)) OR (distance)) OR (travel time)) AND (cancer)) AND ((mortality) OR (survival))) AND (Africa) to identify relevant studies published from database inception to before Oct 16, 2023. No other criteria were used to include or exclude studies. We identified two studies that reported associations between a geospatial characteristic and cancer survival in sub-Saharan Africa, one in Ethiopia (n=302) and one in Nigeria (n=609). Both found that rural residence and travel time to hospital were associated with poorer survival among patients with breast cancer. The smaller study did not report the method for estimating travel time, and both studies constructed their patient cohorts retrospectively, and were likely to have had substantial differential loss to follow-up.

**Added value of this study**

This study provides much needed, high-quality data from sub-Saharan Africa to understand how geospatial characteristics affect the survival of patients with breast cancer. The African Breast Cancer-Disparities in Outcomes study is Africa's largest multicountry cohort of women newly diagnosed with breast cancer. The women were recruited prospectively (2014–17), allowing for the collection of detailed sociodemographic, lifestyle, reproductive, medical, and qualitative data, and were actively followed up every 3 months to ascertain vital status with very low loss to follow-up. We estimated travel time using the latest geospatial analysis methods.

**Implications of all the available evidence**

In a setting with already poor cancer survival, women in sub-Saharan Africa diagnosed with breast cancer face substantial disparities in mortality on the basis of their residential proximity to the treatment hospital. Women living far away in rural areas, in particular, need sustained logistical and financial support to initiate and complete curative treatment regimens, with the greatest survival gains expected in women diagnosed with early-stage disease.

To reduce global breast cancer mortality, particularly by targeting global inequities in survival, WHO launched the Global Breast Cancer Initiative in 2021.^10^ However, in regions such as sub-Saharan Africa where few population-based studies exist, further data are needed to guide its strategies. To identify patient populations vulnerable to poor breast cancer outcomes and where system strengthening is needed to improve breast cancer survival in sub-Saharan Africa, we examined geospatial disparities in 4-year survival in the ABC-DO breast cancer cohort.

## Methods

### Study design and participants

In this prospective cohort study, women aged 18 years or older with suspected breast cancer from eight hospitals located in five sub-Saharan African countries (Namibia, Nigeria, South Africa, Uganda, and Zambia) were recruited between Sept 8, 2014, and Dec 31, 2017. Those with a confirmed histopathological, cytological, or clinical diagnosis of breast cancer were eligible for enrolment in the study. All hospitals in the study were tertiary care hospitals, offered surgery and chemotherapy, and three of the hospitals included the only radiation oncology units in their respective countries (Namibia, Uganda, and Zambia). The catchment areas of the participating hospitals were nationwide, except in Nigeria, which included state and local hospitals, and South Africa, which included regional hospitals. Most patients were recruited from large public hospitals, but some were recruited from a small private clinic in Nigeria. Patients paid for treatment out of pocket in all countries except Namibia and South Africa (appendix 1 p 2).

Upon recruitment, participants completed an interviewer-administered questionnaire that collected information on sociodemographic characteristics, residential urbanisation (eg, rural, village, town, or city), and address of residence. A socioeconomic position score was created on the basis of self-reported assets (ie, home ownership, indoor water, flush toilet, electricity, vehicle, stove, fridge, landline, and bed). Women self-reported their HIV infection status in all study sites except in South Africa, where HIV testing occurred. Treatment information (eg, chemotherapy, endocrine therapy, radiotherapy, and surgery) was abstracted from medical records and ascertained repeatedly during follow-up interviews. All participants provided informed consent either in writing or via thumbprint, witnessed by the interviewer. Ethics approval was obtained from all participating institutions (appendix 1 p 2). Participants also consented to the study team accessing their clinical data, which were abstracted for tumour node metastasis staging and establishing a date of diagnosis (the date of biopsy or cytology was prioritised over date of presentation on the basis of the European Network of Cancer Registries guidelines), and to being contacted regularly for study follow-up. Telephone calls were made every 3 months to participants or their next of kin to assess their vital status. Further details of the study protocol have been published elsewhere.^2^, ^11^

### Geospatial analysis

We geocoded the location of each recruitment hospital and the home addresses the women reported in the baseline questionnaire using gazetteers, such as the National Geospatial-Intelligence Agency GEOnet Names Server database, Open Street Maps, and Google Maps.^4^ For each participant, we examined: (1) their rural versus urban residential status, based on either self-reported categories (ie, rural or village *vs* town or city) or, in South Africa where the recruitment hospital had a smaller regional catchment area, living at least 20 km versus less than 20 km from the hospital; (2) the straight-line (ie, Euclidean) distance (km) between the geocoded home and hospital addresses using the Stata package geodist;^12^ and (3) the estimated travel time (min) from their home to their treatment hospital based on maps of road networks, land use and types (built-up areas, crops, grass, etc), and topography and elevation at 1 km spatial resolution, assuming lowest-cost mode of transportation, using AccessMod 5.7.17 (WHO).^13^ Speeds were assigned to each road class, landcover type, and transport scenario based on previous studies.^14^, ^15^ Details are provided in appendix 1 (p 3–5).

### Statistical analysis

The primary outcome was all-cause mortality. We examined survival from the date of diagnosis until the date of death or censoring (4 years after diagnosis or Jan 1, 2020, whichever came first) using Kaplan–Meier curves and restricted mean survival for the overall cohort and by study site or population and geospatial characteristics.^16^, ^17^, ^18^ We also used Cox proportional hazards regression to estimate hazard ratios (HRs) and 95% CIs for the association between each geospatial characteristic and overall mortality in the first 4 years after diagnosis. All models were stratified by population group: Namibia (non-Black women), Namibia (Black women), Nigeria, Uganda, South Africa, and Zambia. Namibian Black and non-Black women were separated due to strong heterogeneity in cancer survival,^2^ whereas all participants from Uganda, Nigeria, and Zambia were Black and the number of non-Black participants from South Africa (37 [6%]) was insufficient to conduct stratified analysis. To examine the contribution of various covariates on geospatial disparities in mortality, we used crude models and models adjusted for the following variables: age (in years, modelled as a cubic spline); age and stage at diagnosis (I, II, III, or IV); age, stage, and treatment status (time-varying, any treatment received *vs* none); and age, stage, treatment, socioeconomic position (population-specific tertiles), and HIV status.

As differences in rural versus urban mortality might not be uniformly distributed, we explored effect modification by distance (ie, continuous and ≤50 km *vs* >50 km) and travel time to the hospital (ie, continuous and ≤1 h *vs* >1 h). We also examined effect modification by two important prognostic factors: stage at diagnosis (early stage [ie, I/II] *vs* late stage [ie, III/IV]) and whether the woman received any treatment (time-varying, any *vs* none). p values for interaction terms indicated evidence of statistical interaction (p_interaction_<0·10).

We verified linearity assumptions by modelling continuous variables (ie, distance and travel time) using restricted cubic splines and visually inspecting graphs of the predicted HRs. We also verified the proportional hazards assumption for all covariates; for variables in violation (p<0·05), time-dependent HRs were subsequently modelled using 3 df in flexible parametric survival models (Stata package stpm2) with the baseline hazard modelled using 5 df. As a sensitivity analysis of self-reported rural versus urban residence, urbanicity was estimated with a GIS-based approach and analysed in Cox regression models (appendix 1 pp 11–12).

### Role of the funding source

The funders of the study had no role in study design, data collection, data analysis, data interpretation, or writing of the report.

## Results

Of the 2313 women with suspected breast cancer screened for eligibility, 2228 women received a breast cancer diagnosis and were enrolled in the ABC-DO study, of which 2101 were included in this analysis. We excluded: 58 women with potentially recurrent cancer, those who had previously received treatment, or those who had no follow-up; 14 women from minority ethnic groups in South Africa due to small numbers (six Asian women and eight White women); and 55 women with missing geocoded home addresses.

**Figure 1:**
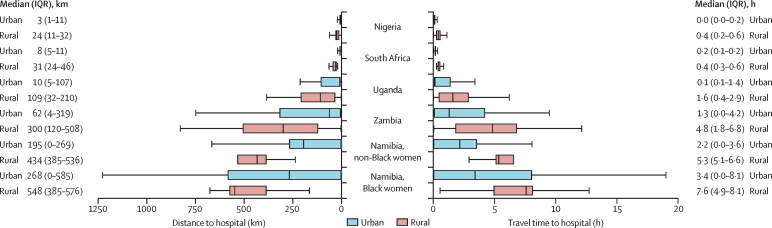
Boxplots of distance (km) and estimated travel time (h) to hospital by study site and rural *vs* urban residence The lines of the box show the first, second, and third quartiles, and the whiskers show the minimum and maximum observed.

Almost half (928 [44%]) of the women lived in a rural area. 47 (4%) of the 1173 participants from urban areas and 25 (3%) from rural areas were lost to follow-up. Compared with women in urban areas, women in rural areas were slightly older (53·1 years [SD 14·5] *vs* 50·5 years [13·7]), were more likely to be Black (904 [97%] of 928 *vs* 1063 [91%] of 1173), and to have ever been married (729 [79%] *vs* 820 [70%]). Women living in rural areas had lower rates of high school education and lived in households with a lower socioeconomic position than women in urban areas (table 1). The straight-line distance from residence to treatment hospital varied widely, with a median of 25·3 km (IQR 6·3–208·2) across all study participants (table 1), and also varied greatly by study population, from a median of 6 km (2–28) in Nigeria to 457 km (194–582) among Black women in Namibia (figure 1). In general, urban dwellers lived closer to the hospital than rural dwellers, although the contrast was minimal in Nigeria and South Africa where most women (340 [90%] in Nigeria and 607 [94%] in South Africa) lived within 50 km (figure 1). Estimated travel times were highly correlated with straight-line distance, with Pearson correlation coefficients within each population group ranging from 0·95 in Nigeria to 0·99 in South Africa. The median estimated travel time was 22 min (IQR 7–166) across all study participants (table 1). Again, women in Nigeria had the shortest median travel time to the treatment hospital (7 min, 2–29), whereas Black women in Namibia had the longest median travel time (5·4 h, 2·2–8·1; figure 1).

**Table 1:** Participant characteristics, overall and by rural *vs* urban residence

		**Rural (n=928)**	**Urban (n=1173)**	**Total (N=2101)**	p value*
Study site or population group		..	..	..	<0·0001
	Namibia, non-Black	9 (9·3%)†	88 (90·7%)†	97 (4·6%)	..
	Namibia, Black	161 (42·5%)†	218 (57·5%)†	379 (18·0%)	..
	Uganda	294 (72·8%)†	110 (27·2%)†	404 (19·2%)	..
	Nigeria	135 (35·6%)†	244 (64·4%)†	379 (18·0%)	..
	South Africa	261 (40·3%)†	386 (59·7%)†	647 (30·8%)	..
	Zambia	68 (34·9%)†	127 (65·1%)†	195 (9·3%)	..
Ethnicity		..	..	..	<0·0001
	Non-Black	24 (2·6%)	110 (9·4%)	134 (6·4%)	..
	Black	904 (97·4%)	1063 (90·6%)	1967 (93·6%)	..
Mean age at diagnosis, years (SD)		53·1 (14·5)	50·5 (13·7)	51·7 (14·1)	<0·0001
Age categories, years		..	..	..	<0·0001
	<40	175 (18·9%)	280 (23·9%)	455 (21·7%)	..
	40 to <50	241 (26·0%)	336 (28·6%)	577 (27·5%)	..
	50 to <60	221 (23·8%)	273 (23·3%)	494 (23·5%)	..
	>60	291 (31·4%)	284 (24·2%)	575 (27·4%)	..
Education level		..	..	..	<0·0001
	Primary school or less	599 (64·5%)	496 (42·3%)	1095 (52·1%)	..
	Secondary or high school	206 (22·2%)	343 (29·2%)	549 (26·1%)	..
	Technical or university	98 (10·6%)	285 (24·3%)	383 (18·2%)	..
	Missing	25 (2·7%)	49 (4·2%)	74 (3·5%)	..
Marital status		..	..	..	<0·0001
	Never married	198 (21·3%)	349 (29·8%)	547 (26·0%)	..
	Divorced or widowed	325 (35·0%)	280 (23·9%)	605 (28·8%)	..
	Married	404 (43·5%)	540 (46·0%)	944 (44·9%)	..
	Missing	1 (0·1%)	4 (0·3%)	5 (0·2%)	..
Mean socioeconomic position score (SD)		3·9 (2·2)	5·8 (1·9)	5·0 (2·2)	<0·0001
Stage at diagnosis		..	..	..	0·0039
	I	35 (3·8%)	81 (6·9%)	116 (5·5%)	..
	II	293 (31·6%)	398 (33·9%)	691 (32·9%)	..
	III	412 (44·4%)	498 (42·5%)	910 (43·3%)	..
	IV	141 (15·2%)	146 (12·4%)	287 (13·7%)	..
	Missing	47 (5·1%)	50 (4·3%)	97 (4·6%)	..
Hormone receptor status					
	ER+	284 (30·6%)	526 (44·8%)	810 (38·6%)	0·0079
	ER data missing	459 (49·5%)	399 (34·0%)	858 (40·8%)	..
	PR+	195 (21·0%)	385 (32·8%)	580 (27·6%)	0·0038
	PR data missing	462 (49·8%)	408 (34·8%)	870 (41·4%)	..
	HER2+	126 (13·6%)	202 (17·2%)	328 (15·6%)	0·86
	HER2 data missing	459 (49·5%)	408 (34·8%)	867 (41·3%)	..
Received any treatment		753 (81·1%)	1002 (85·4%)	1755 (83·5%)	0·0086
Any treatment, by stage‡					
	I	30 (85·7%)	77 (95·1%)	107 (92·2%)	0·084
	II	263 (89·8%)	372 (93·5%)	635 (91·9%)	0·078
	III	343 (83·3%)	430 (86·3%)	773 (84·9%)	0·19
	IV	90 (63·8%)	96 (65·8%)	186 (64·8%)	0·73
	Missing	27 (57·4%)	27 (54·0%)	54 (55·7%)	0·73
Positive HIV status		137 (14·8%)	166 (14·2%)	303 (14·4%)	0·69
Median distance to hospital, km (IQR)		55·0 (25·8–245·7)	8·4 (2·6–80·4)	25·3 (6·3–208·2)	<0·0001
Median travel time to hospital, h (IQR)		0·7 (0·4–3·6)	0·2 (0·0–1·09)	0·4 (0·1–2·8)	<0·0001

Data are presented as mean (SD) or median (IQR) for continuous measures, and n (%) for categorical measures. Socioeconomic position scores are 0 (low) to 9 (high). ER=oestrogen receptor. PR=progesterone receptor.

* p value for comparison between rural and urban data.

† Percentage of total for row.

‡ The denominators are the number of women in each stage, shown in the stage at diagnosis rows.

At the time of this analysis, the median follow-up time was 3·7 years for urban participants and 3·8 years for rural participants (5081·6 total person-years). There were 1042 deaths within 4 years after diagnosis, corresponding to crude overall 4-year survival of 45% (95% CI 42–47), which varied across study populations from 28% (23–33) in Nigeria to 79% (68–86) among non-Black Namibians (appendix 1 p 6).

**Figure 2:**
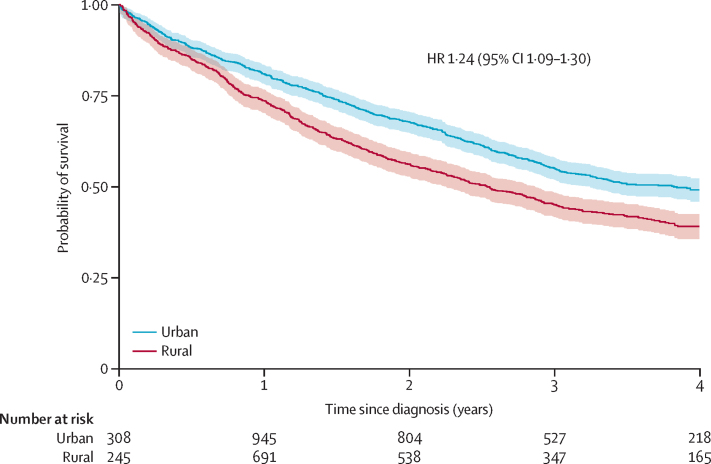
Kaplan–Meier survival curves and 95% CIs for women diagnosed with breast cancer participating in African Breast Cancer-Disparities in Outcomes (n=2101) by rural *vs* urban residence HR=hazard ratio.

Overall, 4-year survival was 10 percentage points lower among women in rural areas (39%, 95% CI 36–42) compared with those in urban areas (49%, 46–52; figure 2; appendix 1 p 6), and their average survival time was 4·1 months (2·5–5·8) shorter (appendix 1 p 9). This finding corresponded to an HR for all-cause mortality among women in rural versus urban areas of 1·24 (95% CI 1·09–1·40), which was not accounted for by differences in age, but stage at diagnosis explained some of the association (table 2; appendix 1 p 7). After additional adjustment for socioeconomic position and HIV status, the latter of which did not differ by rural or urban residence, the mortality rate was no longer different between rural and urban women (HR 1·06, 0·92–1·21; table 2). Further adjustment by distance or travel time did not meaningfully change these estimates (appendix 1 p 7). Similar results were found with the GIS-based rural versus urban definition (fully adjusted HR 1·13, 0·97–1·31; appendix 1 p 12).

**Table 2:** HRs and 95% CIs for all-cause mortality associated with geospatial characteristics in women diagnosed with breast cancer per variable and model

		**HR (95% CI)**	**Stratified HR (95% CI)**	**Stratified HR (95% CI)**	**p**_interaction_
Rural *vs* urban, overall and by distance (>50 km or ≤50 km)		Overall (n=2101)	Distance >50 km (n=815)	Distance ≤50 km (n=1286)	..
	Crude	1·24 (1·09–1·40)	1·46 (1·18–1·81)	1·09 (0·93–1·28)	0·026
	+ Age	1·24 (1·09–1·41)	1·46 (1·18–1·80)	1·10 (0·93–1·29)	0·033
	+ Stage	1·14 (1·00–1·30)	1·29 (1·04–1·61)	1·04 (0·88–1·23)	0·11
	+ Treatment	1·15 (1·00–1·31)	1·29 (1·04–1·62)	1·04 (0·88–1·23)	0·11
	+ Socioeconomic position score and HIV status	1·06 (0·92–1·21)	1·19 (0·94–1·50)	0·98 (0·83–1·16)	0·17
Rural *vs* urban, overall and by travel time (>1 h or ≤1 h)		Overall (n=2101)	Travel time >1 h (n=734)	Travel time ≤1 h (n=1367)	..
	Crude	1·24 (1·09–1·40)	1·49 (1·19–1·87)	1·11 (0·95–1·29)	0·030
	+Age	1·24 (1·09–1·41)	1·49 (1·19–1·87)	1·11 (0·95–1·30)	0·030
	+Stage	1·14 (1·00–1·30)	1·34 (1·06–1·70)	1·05 (0·90–1·24)	0·080
	+Treatment	1·15 (1·00–1·31)	1·35 (1·07–1·71)	1·05 (0·89–1·23)	0·070
	+Socioeconomic position score and HIV status	1·06 (0·92–1·21)	1·23 (0·97–1·57)	0·99 (0·84–1·16)	0·12
Distance (per 50 km), overall and by rural *vs* urban		Overall (n=2101)	Rural (n=928)	Urban (n=1173)	..
	Crude	1·02 (1·01–1·04)	1·04 (1·02–1·07)	1·02 (1·00–1·03)	0·068
	+Age	1·02 (1·01–1·04)	1·04 (1·01–1·07)	1·02 (1·00–1·03)	0·084
	+Stage	1·01 (1·00–1·03)	1·04 (1·01–1·07)	1·01 (0·99–1·03)	0·044
	+Treatment	1·02 (1·00–1·03)	1·04 (1·01–1·07)	1·01 (0·99–1·03)	0·035
	+Socioeconomic position score and HIV status	1·02 (1·00–1·03)	1·03 (1·01–1·06)	1·01 (1·00–1·03)	0·13
Travel time (per 1 h), overall and by rural *vs* urban		Overall (n=2101)	Rural (n=928)	Urban (n=1173)	..
	Crude	1·04 (1·01–1·06)	1·06 (1·02–1·09)	1·02 (1·00–1·05)	0·11
	+Age	1·04 (1·01–1·06)	1·05 (1·02–1·09)	1·02 (1·00–1·05)	0·15
	+Stage	1·03 (1·00–1·05)	1·06 (1·02–1·10)	1·02 (0·99–1·04)	0·038
	+Treatment	1·03 (1·00–1·05)	1·06 (1·02–1·10)	1·02 (0·99–1·04)	0·032
	+Socioeconomic position score and HIV status	1·03 (1·00–1·05)	1·05 (1·01–1·09)	1·02 (0·99–1·05)	0·13

HRs and 95% CIs estimated with Cox proportional hazards models stratified on study site or population (ie, Namibian non-Black women, Namibian Black women, Ugandan women, Nigerian women, South African women, and Zambian women). Stratified HRs were produced from models with interaction terms between the main geospatial variable of interest and the effect modifier. HR=hazard ratio.

Overall, both distance and travel time were also associated with higher mortality rates: HR 1·02 (1·00–1·03) for every additional 50 km, and HR 1·03 (1·00–1·05) for every additional hour of travel time in fully adjusted models (table 2; appendix 1 p 7). Further adjustment by rural versus urban residence did not meaningfully change these estimates (appendix 1 p 7).

Examining rural versus urban residence and distance or time together revealed a survival disadvantage only among women in rural areas living far from the hospital. Among women living more than 1 h from the hospital, crude 4-year overall survival was 37% (95% CI 32–42) in women with rural residence compared with 54% (46–62) in women with urban residence; the mean survival time of rural patients living more than 1 h from the hospital was 6·8 months (95% CI 4·3–9·3) shorter than urban patients living more than 1 h from the hospital (appendix 1 p 9). This finding corresponded to a crude HR of 1·49 (95% CI 1·19–1·87), which was attenuated to 1·35 (1·07–1·71) after adjusting for age, stage, and treatment status, but the 95% CI included 1·0 after adjusting for socioeconomic position and HIV (table 2; appendix 1 p 8). Results were similar among women living more than 50 km from the hospital (table 2; appendix 1 p 8). In contrast, among women living within 1 h or 50 km of the hospital, there was no evidence of increased mortality of women in rural versus urban areas. Interactions with continuous distance or travel time were also significant (p_interaction_ values ranging across models from 0·0055 for crude distance and 0·031 for fully adjusted travel time). Plots of the predicted HRs for rural versus urban residence over distance or travel time are shown in appendix 1 (p 8).

Similarly, increasing mortality rates associated with distance or travel time were observed among women in rural areas (eg, per 1 h travel time, fully adjusted HR 1·05, 95% CI 1·01–1·09), but not among women in urban areas (eg, per 1 h travel time, fully adjusted HR 1·02, 0·99–1·05; table 2; appendix 1 p 8). With the GIS-based rural versus urban definition to examine effect modification, associations were attenuated but still present only among women in rural areas (appendix 1 p 12).

**Figure 3:**
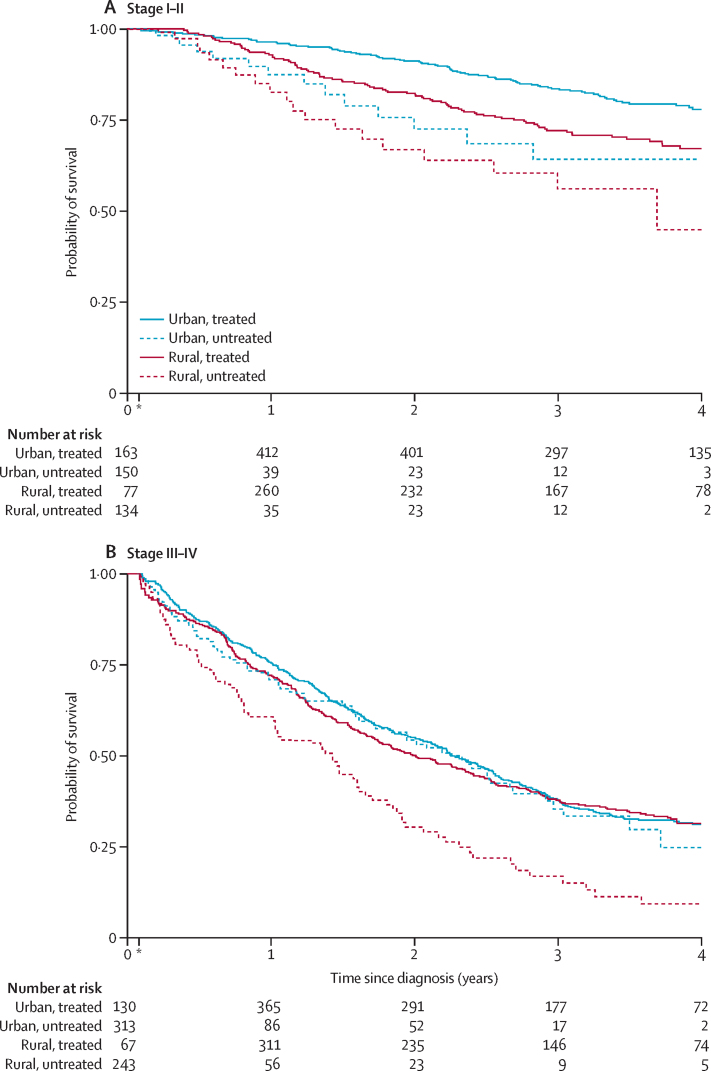
Kaplan–Meier survival curves for women diagnosed with breast cancer participating in ABC-DO (n=2101) by rural or urban residence and time-varying treatment status (A) Women with breast cancer diagnosed at early stages (I/II). (B) Women with breast cancer diagnosed at late stages (III/IV). ABC-DO=African Breast Cancer-Disparities in Outcomes. *Curves and risk tables begin at 1 month due to small numbers.

The survival disadvantage of women in rural areas differed strongly by tumour stage at diagnosis and whether they received any treatment (p_interaction_ value ranged from 0·034 to 0·042 across the four models; table 3; figure 3). There was a clear survival disadvantage among women from rural versus urban areas diagnosed at an early stage who received treatment (fully adjusted HR 1·42, 1·05–1·92), which increased further when restricted to women living more than 1 h (fully adjusted HR 2·17, 1·21–3·89) or more than 50 km (fully adjusted HR 2·06, 1·19–3·59) from the hospital (table 3). In the untreated early-stage group, mortality was not significantly higher among women from rural versus urban areas. This finding is expected if difficulties in adhering to treatment regimens explain why women from rural areas have higher mortality than women from urban areas. Among women diagnosed at a late stage (ie, III/IV) who received treatment, women living in rural and urban areas had similar mortality rates, but after restricting to those living more than 50 km or more than 1 h from the hospital, a clear survival disadvantage was seen for women living in rural versus urban areas, although CIs were wide (table 3). In the untreated late-stage group, women living in rural versus urban areas also had worse survival (table 3).

**Table 3:** HRs and 95% CIs for all-cause mortality associated with women with breast cancer of self-reported rural *vs* urban residence by stage at diagnosis and treatment status per group and model

		**Stage I/II, treated**	**Stage I/II, untreated**	**Stage III/IV, treated**	**Stage III/IV, untreated**	**p**_interaction_
Rural *vs* urban		n=742	n=65	n=959	n=238	..
	Crude	1·54 (1·14–2·07)	1·21 (0·62–2·37)	1·46 (0·81–2·61)	1·72 (1·30–2·26)	0·034
	+ Age	1·55 (1·16–2·09)	1·23 (0·63–2·40)	1·47 (0·82–2·63)	1·69 (1·28–2·23)	0·036
	+ Stage	1·54 (1·14–2·07)	1·22 (0·62–2·38)	1·82 (1·01–3·27)	1·64 (1·24–2·15)	0·042
	+ Socioeconomic position score and HIV status	1·42 (1·05–1·92)	1·13 (0·57–2·23)	1·64 (0·90–2·99)	1·55 (1·18–2·05)	0·035
Rural *vs* urban in >50 km subgroup		n=280	n=18	n=407	n=66	..
	Crude	2·06 (1·21–3·53)	1·22 (0·32–4·55)	4·38 (1·13–16·94)	2·54 (1·35–4·79)	0·10
	+ Age	2·11 (1·23–3·61)	1·19 (0·32–4·48)	4·46 (1·15–17·28)	2·51 (1·33–4·74)	0·091
	+ Stage	2·08 (1·21–3·58)	1·08 (0·29–4·06)	5·66 (1·45–22·04)	2·34 (1·23–4·44)	0·075
	+ Socioeconomic position score and HIV status	2·06 (1·19–3·59)	1·06 (0·28–4·02)	5·48 (1·40–21·49)	2·36 (1·22–4·55)	0·072
Rural *vs* urban in >1 h subgroup		n=252	n=15	n=376	n=55	..
	Crude	2·13 (1·21–3·73)	1·27 (0·33–4·85)	4·51 (1·14–17·80)	2·61 (1·34–5·08)	0·10
	+ Age	2·17 (1·23–3·81)	1·26 (0·33–4·83)	4·67 (1·18–18·45)	2·66 (1·36–5·21)	0·092
	+ Stage	2·14 (1·21–3·78)	1·13 (0·29–4·34)	6·21 (1·55–24·83)	2·69 (1·36–5·31)	0·063
	+ Socioeconomic position score and HIV status	2·17 (1·21–3·89)	1·14 (0·29–4·41)	6·09 (1·51–24·55)	2·79 (1·38–5·64)	0·061

Data are HR (95% CI) unless otherwise stated. Cox proportional hazards models stratified on study site and population (ie, Namibian non-Black women, Namibian Black women, Ugandan women, Nigerian women, South African women, and Zambian women), with three-way interaction between rural *vs* urban residence, early (ie, I and II) *vs* late (ie, III and IV) stage at diagnosis, and time-varying treatment status (any *vs* none). HR=hazard ratio.

Because the rural versus urban effect violated the proportional hazards assumption (p values for the proportional hazards violation ranged from 0·0046 to 0·011), we also fitted flexible parametric survival models that allow the HR to change over time. In fully adjusted models, the time-dependent ratio of mortality rates among women of rural versus urban residence was highest at 0·9 years after diagnosis (HR_peak_ 1·30, 95% CI 1·09–1·54) and was elevated from 0·5 years to 1·5 years after diagnosis (appendix 1 p 10). Among women living more than 50 km from the hospital, the ratio was highest at 1·0 year after diagnosis (HR_peak_ 1·75, 1·25–2·44) and was elevated from 0·4 years to 2·2 years after diagnosis (appendix 1 p 10).

## Discussion

In this large, multicountry prospective cohort of women diagnosed with breast cancer in sub-Saharan Africa, we identified a clear and substantial survival disadvantage among women living in rural areas far from the treatment hospital (ie, >50 km or >1 h), who died at a higher rate than women living equally far away but in urban areas. We previously showed that women in rural areas are diagnosed at later stages than women living in urban areas,^4^ thus a crude survival disadvantage was expected. However, it was not fully explained by differences in age, stage, or even whether they had received any treatment. That the 95% CI included 1·0 after further adjustment for socioeconomic position—and HIV, which did not differ by rural versus urban residence—signals the importance of financial resources for cancer survival, most likely through enabling patients to complete treatment regimens that are paid for out-of-pocket in most settings. The survival disadvantage of women in rural areas was strong and consistent among those receiving treatment for early-stage disease and was also evident among women living far from the treatment hospital (ie, >50 km or >1 h) and receiving treatment for late-stage disease. This finding indicates that even when women in rural areas overcome barriers to early diagnosis^4^ and begin potentially curative treatment, they still face additional challenges in effective disease management compared with those living in urban areas.

Our study is the first multicountry study to examine geospatial determinants of cancer survival in sub-Saharan Africa. Although some studies have reported greater delays in cancer diagnosis with increasing distance or travel time in these settings,^5^, ^6^, ^7^, ^8^ two studies previously examined the effect on cancer outcomes. In a retrospective cohort of 302 patients with breast cancer from two major cancer referral centres for southern Ethiopia, rural residence (HR 2·71, 95% CI 1·44–5·09) and travel time exceeding 7 h (3·42, 1·05–11·10) were associated with mortality.^9^ The methods for determining rural or urban residence and travel time were not specified, and 19% of the cohort was lost to follow-up, with substantial uncertainty in the date of censoring or death.^9^ In a cohort of 609 patients with breast cancer constructed, at least in part, retrospectively at a tertiary referral centre for Osun state in Nigeria, GIS-based travel time exceeding 30 min was associated with an HR of 1·65 (1·17–1·33), adjusting for age, education, and socioeconomic position.^8^ This association is stronger than our data suggest, which could be explained by shorter median follow-up time (1 year *vs* 3·7 years in ABC-DO) that started at hospital presentation (*vs* diagnosis in ABC-DO), as well as biases related to loss to follow-up and misclassification in the date of censoring. Nevertheless, all three studies agree that greater travel time reduces the survival of women with breast cancer in sub-Saharan Africa.

The current standard of multimodality breast cancer treatment in sub-Saharan Africa typically involves surgery, often after neoadjuvant chemotherapy, or endocrine treatment and radiotherapy, or a combination of these treatments. These therapies require repeated treatment and follow-up visits over a period of many months. Data from high-income countries suggest that receipt of guideline-discordant treatment might be an important pathway linking excess travel burden to poor survival outcomes.^19^ We previously reported very low concordance with treatment guidelines set by the WHO Global Breast Cancer Initiative within ABC-DO;^10^, ^20^ less than half (45%) of participants completed their recommended cycles of chemotherapy in a 6-month period.^20^ Greater delays in treatment initiation, lower adherence to treatment schedules, and greater treatment abandonment are potential explanations for the worse prognosis of women facing geospatial barriers.

This study is the first multicountry prospective study to describe geospatial disparities in mortality of women with breast cancer in sub-Saharan Africa. The ABC-DO study is unique in its prospective design and use of mobile health to conduct follow-up of a cohort of women with incident breast cancer, along with the scope and breadth of information collected from direct patient interview, allowing for the collection of detailed sociodemographic, lifestyle, reproductive, medical, and qualitative data. There was also very low loss to follow-up (72, 3·4%), facilitated by regular telephone calls to participants and their next of kin at 3-month intervals, which also allowed for accurate date of death ascertainment.^21^ The multicountry design highlights the importance of context because we have often found a great degree of heterogeneity between countries.^2^, ^4^, ^20^, ^22^ Furthermore, the methods we used to estimate travel time to the treatment hospital are the industry standard for modelling spatial accessibility, improve upon measures such as straight-line distance and distance to the nearest hospital, and are considered more accurate than self-reported travel time. Some measurement error is expected due to reporting of the nearest landmark (eg, police station, bus stop, farm, or compound), district, or even village in the absence of formal addresses, although the error might be small in relation to the vast distances or travel time, and the direction of bias in the HR is difficult to predict.^23^

We examined all-cause mortality because cause-specific mortality reported by next of kin is not reliable and national death registries were not available. However, most deaths were likely to be breast cancer-related: mortality was lower for women with breast cancer diagnosed at earlier stages and who were treated, and the mortality rate in untreated women was in line with expected rates for untreated breast cancer.^24^ As observed in the early-stage group, no geospatial differences in mortality are expected among untreated women after adjusting for stage. The higher HRs for rural residence than for urban residence among untreated women with late-stage disease could be explained by more advanced disease (eg, stage IIIB or IIIC *vs* IIIA) and greater delays in treatment initiation among women in rural areas. Finally, adjusting for tumour hormone-receptor status would have improved statistical efficiency, but these data were missing for many women—more often for women in rural areas—and did not vary by distance or travel time (data not shown), therefore are unlikely to explain our observed associations.

Women diagnosed with breast cancer in sub-Saharan Africa have numerous challenges in reaching and interacting with the health system.^25^ Hospitals with specialised cancer care facilities are located in major cities, whereas more than half (58%) of the population of sub-Saharan Africa live in rural areas.^26^ The vast geographical area, combined with poor road infrastructure and transportation services, mean that some women travel hundreds of kilometres over multiple days to reach the hospital, incurring expenses related to travel and accommodation, childcare, and lost wages, all in addition to high out-of-pocket medical costs because of the absence of universal health coverage in most of sub-Saharan Africa.^25^, ^27^, ^28^

Nevertheless, the rapid expansion of cancer treatment centres in many sub-Saharan African settings in the past decade (ie, 2010s) will reduce these barriers, so that fewer women will live so far from a treatment centre. For example, since the ABC-DO recruitment period (2014–2017), chemotherapy is now available in Namibia at two locations outside of the capital. To address geographical inequities in access to treatment, the *Lancet Oncology* Commission on cancer in sub-Saharan Africa^25^ proposes chemotherapy to be decentralised into regional centres. The Butaro Cancer Center of Excellence in northern Rwanda might be one model for providing rural cancer care in sub-Saharan Africa, offering free cancer services including diagnosis, staging, chemotherapy, referral for imaging, radiotherapy and surgery, and follow-up, as well as mental health services, social workers, meals, transportation, and home visits.^29^ Other potential solutions include mobile units equipped to provide diagnosis and treatment using modern technological platforms (eg, telepathology and telesurgery). Any interventions intended to reduce geospatial disparities in breast cancer survival should be carefully considered and adapted to each setting and properly evaluated before widespread implementation. Broader changes to strengthen the cancer control system in sub-Saharan Africa, such as the removal of patient financial burden through universal health coverage, are also needed.^25^

Patients with breast cancer living in rural areas in sub-Saharan Africa living far from the treatment hospital have worse survival than patients living in urban areas, especially those undergoing treatment for early-stage disease. These findings indicate that substantial barriers exist not only in access to timely diagnosis, which we had previously reported,^4^ but also in accessing effective disease management and treatment once they are diagnosed. Providing specific support to such patients, to empower and enable them to complete curative treatment regimens for breast cancer, might help to avert breast cancer deaths in these women.

## Equitable partnership declaration

## Data sharing

The data collected as part of the ABC-DO study are described in our open-access study protocol (https://doi.org/10.1136/bmjopen-2016-011390). Data will be made available upon reasonable request to the principal investigators (VM [mccormackv@iarc.who.int] and IdS-S [isabel.silva@lshtm.ac.uk]). Reasonable access criteria include research proposals for stand-alone analysis within the scope of this study. Such proposals will be evaluated and supported if deemed relevant. Proposals from researchers and doctoral students in low-income and middle-income countries are encouraged. Relevant data will be shared with a signed data access agreement.

## Declaration of interests

JK is supported by a postdoctoral award from the Fonds de Recherche du Québec–Santé. MG is a THRiVE-2 fellow (DELTA African Initiative DEL-15-011). LFP is supported by the University of Washington (Seattle, WA, USA) T32 Fellowship (5T32CA009515-34). All other authors declare no competing interests.

## Supplementary Materials

Supplementary appendix 1

Equitable Partnership Declaration
