# Supplementary material for: Geospatial disparities in survival of patients with breast cancer in sub-Saharan Africa from the African Breast Cancer-Disparities in Outcomes cohort (ABC-DO): a prospective cohort study
Source: Lancet Glob Health. 2024 May 21;12(7):e1111–9. doi: 10.1016/S2214-109X(24)00138-4 (PMC11168938; doi:10.1016/S2214-109X(24)00138-4)
Supplement: Supplementary appendix 1 [file mmc1.pdf]

THE LANCET
Global Health

Supplementary appendix 1

This appendix formed part of the original submission and has been peer reviewed. We post it as supplied by the authors.

Supplement to: Kim J, Macharia PM, McCormack V, et al. Geospatial disparities in survival of patients with breast cancer in sub-Saharan Africa from the African Breast Cancer-Disparities in Outcomes cohort (ABC-DO): a prospective cohort study. *Lancet Glob Health* 2024; published online May 21. https://doi.org/10.1016/S2214-109X(24)00138-4.

TABLE OF CONTENTS

I. STUDY DESIGN 2

Table S1. ABC-DO Recruitment hospitals 2

II. GIS METHODS: ESTIMATING TRAVEL TIME 3

Figure S1. GIS data used in the calculation of travel times (example: Uganda and neighbouring countries) 4

III. SURVIVAL ANALYSES 6

Table S2. Crude 4-year survival rates, overall and by rural vs. urban, stage at diagnosis, and study site/population 6

Figure S2. Kaplan-Meier survival curves for women diagnosed with breast cancer participating in ABC-DO (n=2101), by rural (orange) vs. urban (blue) residence, separate by study site/population . 6

Figure S3. Hazard ratios (HRs) and 95% confidence intervals (CIs) for self-reported rural vs. urban residence in crude and adjusted models [orange], with and without additional adjustment for distance [grey] and travel time [black] 7

Figure S4. Hazard ratios (HRs) and 95% confidence intervals (CIs) for distance and travel time, with and without additional adjustment by self-reported rural vs. urban residence 7

Figure S5. Hazard ratios (HR) and 95% confidence intervals (CI) for all-cause mortality associated with self-reported rural vs. urban residence, by: a) distance (≤ or >50 km), b) travel time (≤ or >1 hr), c) continuous distance (km), or d) continuous travel time (hr) 8

Figure S6. Hazard ratios (HRs) and 95% confidence intervals (CIs) for distance and travel time, stratified by self-reported rural vs. urban residence 8

Table S3. Restricted mean survival time and 95% confidence intervals (CI) in rural vs. urban women diagnosed with breast cancer in the ABC-DO cohort study 9

Table S4. Time-dependent HRs for self-reported rural vs. urban residence, overall and among women living ≥50km from the hospital 10

Figure S7. Time-dependent HRs for self-reported rural vs. urban residence, overall and among women living ≥50km from the hospital 10

IV. SENSITIVITY ANALYSIS: GIS-BASED RURAL VS. URBAN 11

Figure S8. Proportion of self-reported residential areas classified as rural (mean GIS urbanicity score <4) using GIS-based methods 11

Table S5. Hazard ratios (HR) and 95% confidence intervals (CI) for all-cause mortality associated with geospatial characteristics, using the GIS-based rural vs. urban residence, in women diagnosed with breast cancer in the ABC-DO cohort study 12

I. STUDY DESIGN

List of ethics approvals obtained for the ABC-DO study

1. IARC (IEC13-19, IEC15-18)
2. LSHTM (6459)
3. Federal Medical Centre Owerri, Nigeria
4. Abia State University Teaching Hospital, Nigeria
5. University of Zambia Biomedical Research Ethics Committee (004-08-15), Zambia
6. University of Witwatersrand (M150345), Gauteng, South Africa
7. Uganda National Council for Science and Technology (HS 1588), Uganda
8. Ministry of Health and Social Services of Namibia (17/3/3)

Table S1. ABC-DO Recruitment hospitals

Country	ABC-DO Recruitment Hospitals (City)	Catchment	Sector	Treatment costs to patient
Namibia	Windhoek Central Hospital (Windhoek)	National	Public	Free/minimal
Nigeria	Abia State University Teaching Hospital (Aba)<br>Federal Medical Centre (Owerri)<br>Maranatha private clinic (Aba)	State-wide<br>State-wide<br>Local	Public<br>Public<br>Private	Out-of-pocket
South Africa	Chris Hani Baragwanath Academic Hospital (Soweto)	Regional	Public	Low
Uganda	Mulago Hospital (Kampala)	National	Public	Out-of-pocket
Zambia	Cancer Diseases Hospital (Lusaka)<br>Kabwe General Hospital (Kabwe)	National<br>Regional	Public	Out-of-pocket

II. GIS METHODS: ESTIMATING TRAVEL TIME

Data assembly

a. *Road network*. We assembled the latest road network for each country from OpenStreetMap [1]. The road network was reclassified into four classes, namely primary, secondary, tertiary, and minor, based on the road attributes data from OpenStreetMap (see **Figure S1a**).[1]
b. *Land use:* We used land cover data in areas where there was either no road network or data from OpenStreetMap might have been incomplete. The landcover information was based on Sentinel-2 satellite at 10 by 10 metres spatial resolution (see **Figure S1b**).[2,3]
c. *Travel barriers*. The barriers to travel considered were water bodies and protected areas. They were considered impassable except in the presence of a bridge where a road intersected a water body (see **Figure S1b**).[4]
d. *Digital elevation model:* The slope of the land impedes walking and was obtained from Shuttle Radar Topographic Mission Digital elevation models at the 30m x 30m resolution.[5,6] The walking speeds were corrected according to Tobler's formulation, an exponential function that describes how human walking speed varies with slope (see **Figure S1c**).[4,7]

Modelling travel times

To model travel time between the residence of each participant to the hospital they attended, we used approaches that have been widely used to model healthcare accessibility across sub-Saharan Africa.[8-10] For example, in Tanzania,[11] Ethiopia,[12] Uganda,[13] Rwanda,[14] Ghana,[15] Niger,[16] Sierra Leone,[17,18] Togo,[19] Mozambique[20] and Namibia.[21] Specifically, in order to compute travel time, a least-cost path algorithm that minimizes the total travel duration between each patient's residential location and hospital was implemented. This is distinct from an Euclidean distance because the least cost path accounts for the mode of transport and corresponding travel speeds used by each patient, including travel barriers.

The algorithm was implemented using AccessMod 5.7.17 (WHO, Geneva, Switzerland), a free, open-source, user-friendly tool supported by the World Health Organization (WHO) to analyse geographic accessibility.[7,22] AccessMod imports user-provided geospatial datasets (see Data Assembly section) to informs the parameters required to calculate the travel time to the hospital.[7,22]

First the *"merge land cover module"* in AccessMod 5.7.17 (WHO, Geneva, Switzerland), was used to overlay and merge the road network, landcover, water bodies and protected areas to obtain a single raster dataset to which different modes of transport were applied based on the patient records. Second, the least cost path (cost measured in terms of time) was invoked in the "*accessibility module"* of AccessMod 5.7.17 (WHO, Geneva, Switzerland) to compute the cumulative travel time from residences to the utilised facility by bringing together the merged layer, hospitals and travel speeds (see **Figure S1d**). The travel speeds assigned to each road class, landcover type and travel scenario were based on a review of from previous comparable studies.[8,23] The analysis was conducted at 1 km spatial resolution considering one country (and its neighbouring countries where recruited patients resided) at a time.

Figure S1. GIS data used in the calculation of travel times (example: Uganda and neighbouring countries)

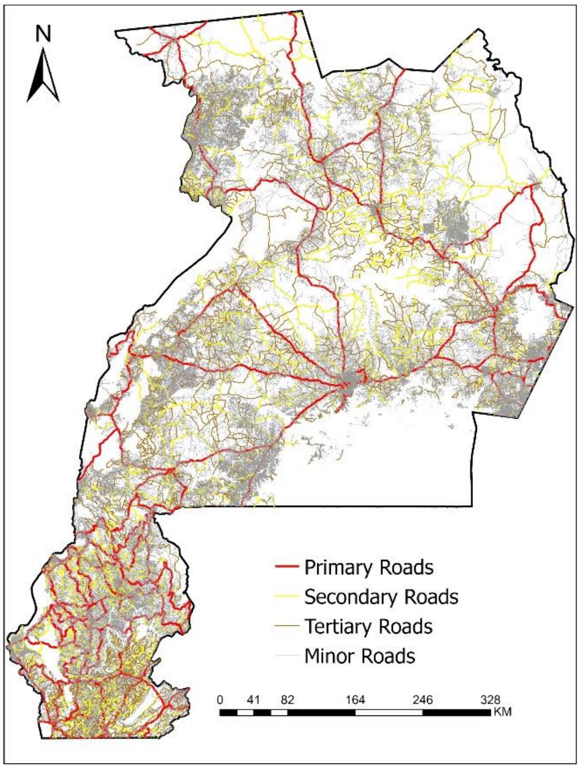

a) Road networks

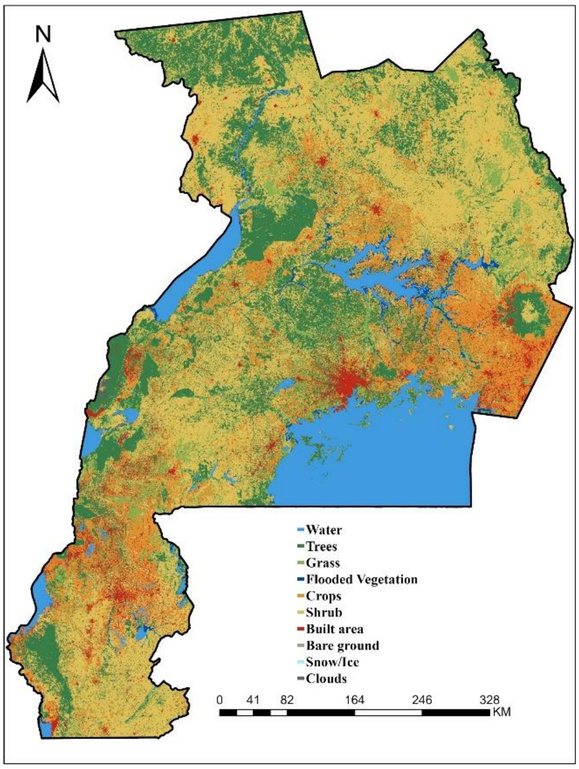

b) Land use and land cover, including travel barriers

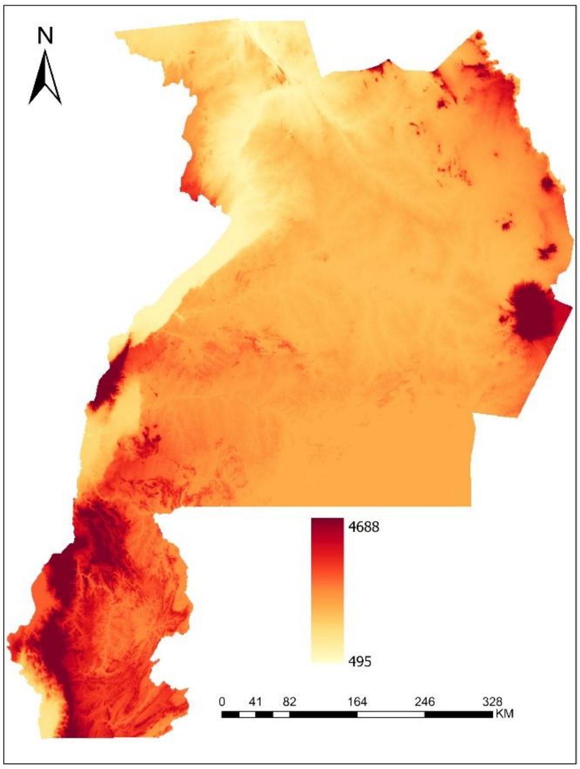

c) Digital elevation model

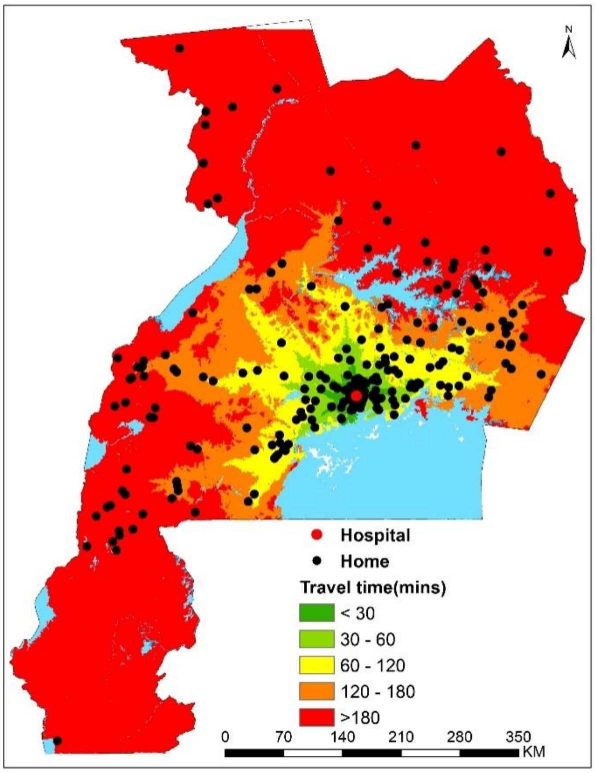

d) Resulting modelled travel time estimates

Section II References

1. Geofabrik GmbH. Download OpenStreetMap data for Africa. 2023. https://download.geofabrik.de/africa.html (accessed 9 December 2023)
2. Karra K, Kontgis C, Statman-Weil Z, *et al.* Global land use/land cover with Sentinel-2 and deep learning. *IGARSS 2021-2021 IEEE International Geoscience and Remote Sensing Symposium*. Brussels: IEEE 2021. https://igarss2021.com/view_paper.php?PaperNum=3500 (accessed 14 November 2021)
3. Phiri D, Simwanda M, Salekin S, *et al.* Sentinel-2 data for land cover/use mapping: A review. Remote Sens (Basel). 2020;12. https://doi.org/10.3390/rs12142291
4. UNEP-World Conservation Monitoring Centre (WCMC), International Union for Conservation of Nature (IUCN). The world database on protected areas. 2023. https://www.protectedplanet.net/en/about (accessed 9 December 2023)
5. Tobler W. Three presentations on geographical analysis and modeling: Non-isotropic geographic modeling speculations on the geometry of geography global spatial analysis. *Technical report (National center for geographic information and analysis)*. 1993.
6. Van Zyl JJ. The shuttle radar topography mission (SRTM): A breakthrough in remote sensing of topography. *Acta Astronautica*. 2001. https://doi.org/10.1016/S0094-5765(01)00020-0
7. Ray N, Ebener S. AccessMod 3.0: Computing geographic coverage and accessibility to health care services using anisotropic movement of patients. *Int J Health Geogr*. 2008;7. doi: 10.1186/1476-072X-7-63
8. Ouma PO, Maina J, Thuranira PN, *et al.* Access to emergency hospital care provided by the public sector in sub-Saharan Africa in 2015: a geocoded inventory and spatial analysis. *Lancet Glob Health*. 2018;6:e342–50.
9. Juran S, Broer PN, Klug SJ, *et al.* Geospatial mapping of access to timely essential surgery in sub-Saharan Africa. *BMJ Glob Health*. 2018;3:e000875.
10. Wigley AS, Tejedor-Garavito N, Alegana V, *et al.* Measuring the availability and geographical accessibility of maternal health services across sub-Saharan Africa. *BMC Med*. 2020;18. doi: 10.1186/s12916-020-01707-6
11. Macharia PM, Beňová L, Pinchoff J, *et al.* Neonatal and perinatal mortality in the urban continuum: a geospatial analysis of the household survey, satellite imagery and travel time data in Tanzania. *BMJ Glob Health*. 2023;8:e011253.
12. Kibret GD, Demant D, Hayen A. Geographical accessibility of emergency neonatal care services in Ethiopia: analysis using the 2016 Ethiopian Emergency Obstetric and Neonatal Care Survey. *BMJ Open*. 2022;12. doi: 10.1136/bmjopen-2021-058648
13. Ouma P, Macharia PM, Okiro E, *et al.* Methods of Measuring Spatial Accessibility to Health Care in Uganda. 2021:77–90. https://doi.org/10.1007/978-3-030-63471-1_6
14. Huerta Munoz U, Källestål C. Geographical accessibility and spatial coverage modeling of the primary health care network in the Western Province of Rwanda. *Int J Health Geogr*. 2012;11. doi: 10.1186/1476-072X-11-40
15. Dotse-Gborgbortsi W, Nilsen K, Ofosu A, *et al.* Distance is "a big problem": a geographic analysis of reported and modelled proximity to maternal health services in Ghana. *BMC Pregnancy Childbirth*. 2022;22. doi: 10.1186/s12884-022-04998-0
16. Oliphant NP, Ray N, Bensaid K, *et al.* Optimising geographical accessibility to primary health care: A geospatial analysis of community health posts and community health workers in Niger. *BMJ Glob Health*. 2021;6. doi: 10.1136/bmjgh-2021-005238
17. Van Duinen AJ, Adde HA, Fredin O, *et al.* Travel time and perinatal mortality after emergency caesarean sections: An evaluation of the 2-hour proximity indicator in Sierra Leone. *BMJ Glob Health*. 2020;5. doi: 10.1136/bmjgh-2020-003943
18. Oliphant NP, Ray N, Curtis A, *et al.* Optimising scale and deployment of community health workers in Sierra Leone: A geospatial analysis. *BMJ Glob Health*. 2022;7. doi: 10.1136/bmjgh-2021-008141
19. Curtis A, Monet JP, Brun M, *et al.* National optimisation of accessibility to emergency obstetrical and neonatal care in Togo: A geospatial analysis. *BMJ Open*. 2021;11. doi: 10.1136/bmjopen-2020-045891
20. Hierink F, Rodrigues N, Muñiz M, *et al.* Modelling geographical accessibility to support disaster response and rehabilitation of a healthcare system: An impact analysis of Cyclones Idai and Kenneth in Mozambique. *BMJ Open*. 2020;10. doi: 10.1136/bmjopen-2020-039138
21. Alegana VA, Wright JA, Pentrina U, *et al.* Spatial modelling of healthcare utilisation for treatment of fever in Namibia. *Int J Health Geogr*. 2012;11. doi: 10.1186/1476-072X-11-6
22. GeoHealth group (University of Geneva), World Health Organization, MORU/Health GeoLab Group. AccessMod 5. 2023. https://www.accessmod.org/ (accessed 25 May 2023)
23. Bouanchaud P, MacHaria PM, Demise EG, *et al.* Comparing modelled with self-reported travel time and the used versus the nearest facility: modelling geographic accessibility to family planning outlets in Kenya. *BMJ Glob Health*. 2022;7. doi: 10.1136/bmjgh-2021-008366.

III. SURVIVAL ANALYSES

Table S2. Crude 4-year survival rates, overall and by rural vs. urban, stage at diagnosis, and study site/population

	Survival rate (95% CI)
**Overall**	45% (42-47%)
**Rural vs. urban**	
Rural	39% (36-42%)
Urban	49% (46-52%)
**Stage**	
I	74% (64-81%)
II	71% (67-75%)
III	35% (31-38%)
IV	7% (5-11%)
**Site/population**	
Namibia, non-Black	79% (68 - 86%)
Namibia, Black	52% (47 - 58%)
Nigeria	28% (23 - 33%)
South Africa	51% (46 - 56%)
Uganda	37% (32 - 42%)
Zambia	42% (34 - 50%)

Figure S2. Kaplan-Meier survival curves for women diagnosed with breast cancer participating in ABC-DO (n=2101), by rural (orange) vs. urban (blue) residence, separate by study site/population

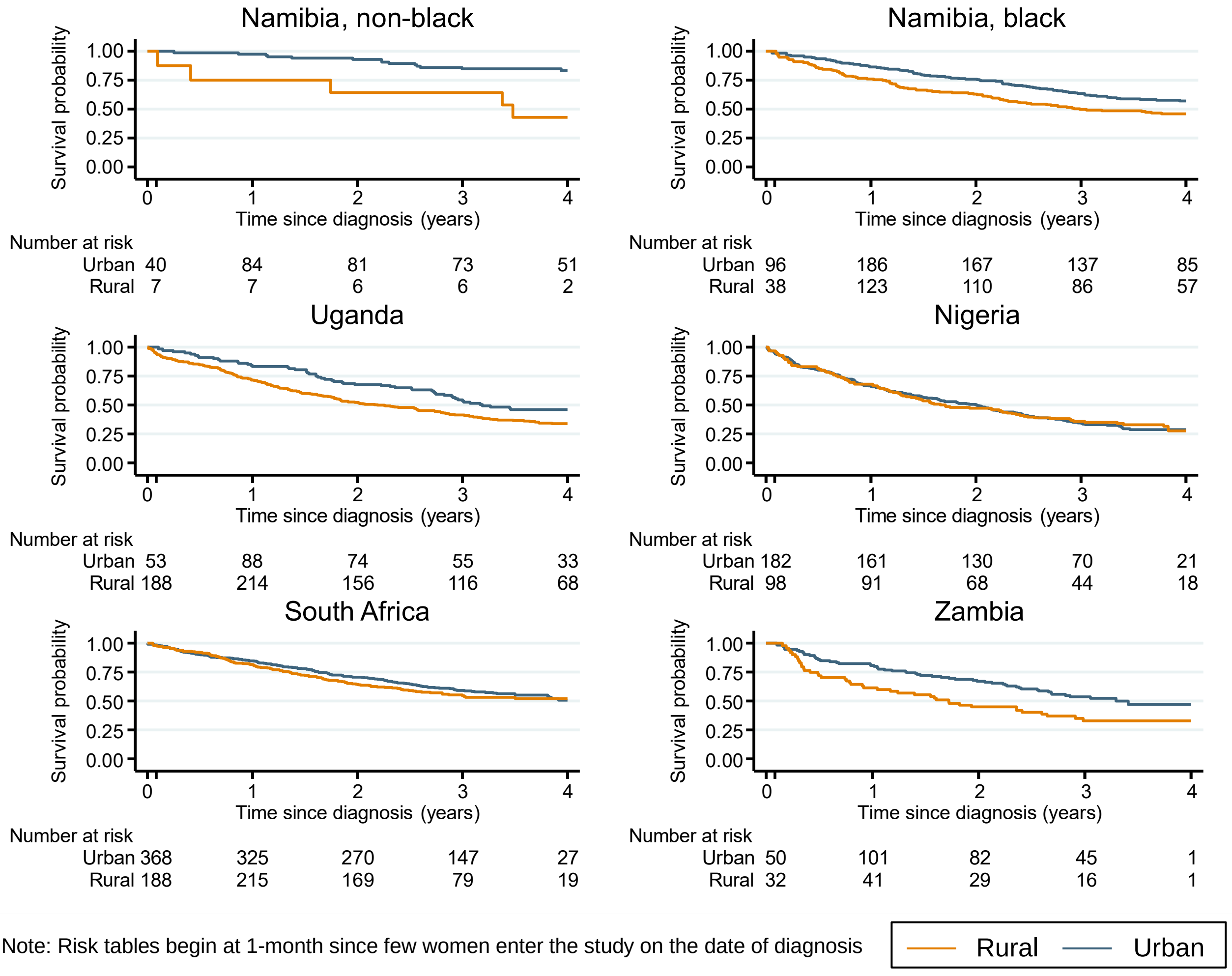

Figure S3. Hazard ratios (HRs) and 95% confidence intervals (CIs) for self-reported rural vs. urban residence in crude and adjusted models [orange], with and without additional adjustment for distance [grey] and travel time [black]

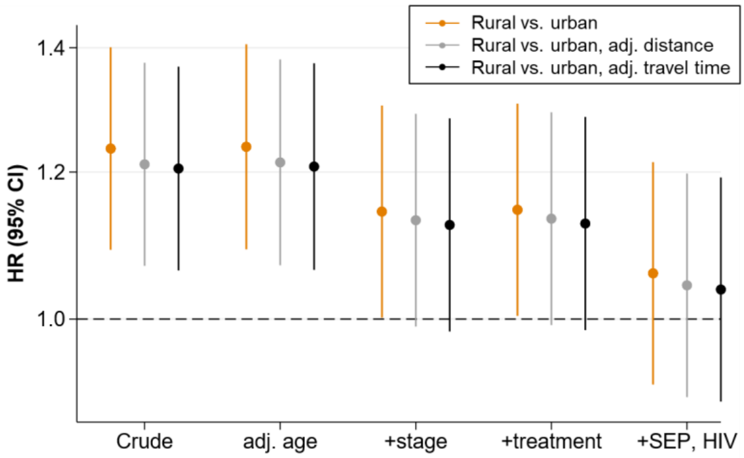

Figure S4. Hazard ratios (HRs) and 95% confidence intervals (CIs) for distance and travel time, with and without additional adjustment by self-reported rural vs. urban residence

a) Distance to hospital (per 50km)

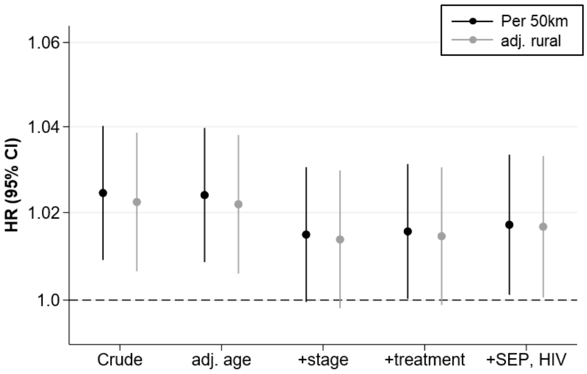

b) Travel time (per hour)

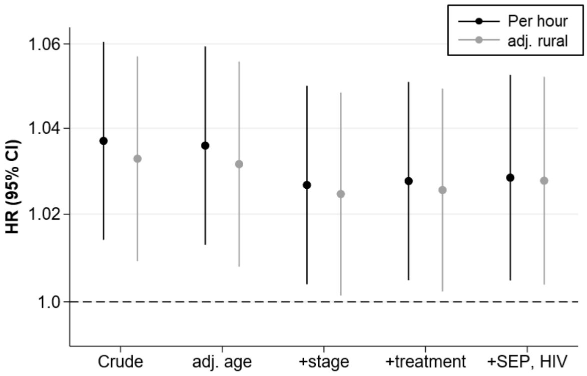

Figure S5. Hazard ratios (HR) and 95% confidence intervals (CI) for all-cause mortality associated with self-reported rural vs. urban residence, by: a) distance (≤ or >50 km), b) travel time (≤ or >1 hr), c) continuous distance (km), or d) continuous travel time (hr)

a) Rural vs. urban, by distance (≤ or >50 km)

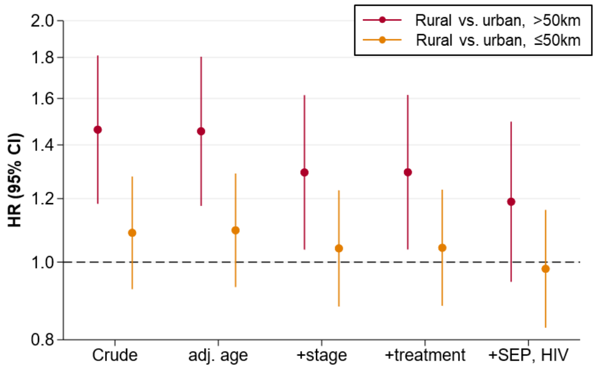

b) Rural vs. urban, by travel time (≤ or >1 hr)

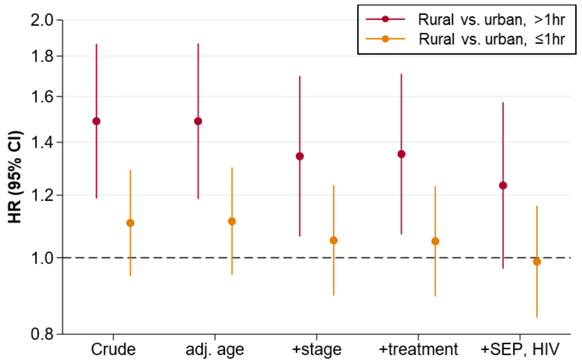

c) Rural vs. urban, by distance (km, continuous)

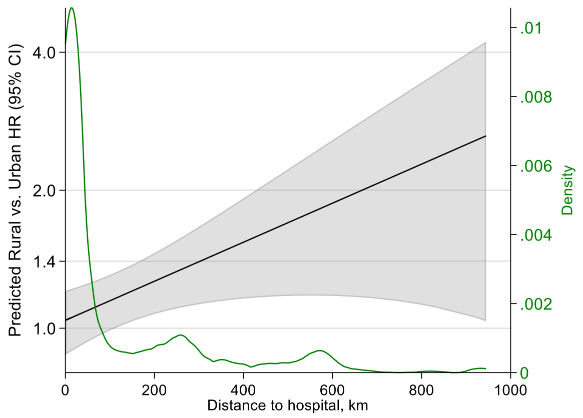

d) Rural vs. urban, by travel time (hr, continuous)

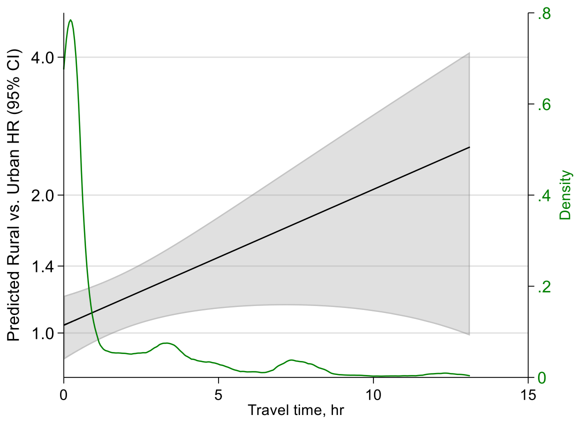

Note: Figures S5c and S5d were generated from Cox models with interaction terms between rural and distance (km) or travel time (hr) expressed continuously, stratified on study site/population and adjusted for age as a spline; outliers (distance >1000km) were excluded due to the strong influence of extreme values. Across all models (crude, adjusted for age, stage, treatment, SEP & HIV), the p-value for interaction ranged from 0.01 to 0.03 for both rural x distance (km) and rural x travel time (hr).

Figure S6. Hazard ratios (HRs) and 95% confidence intervals (CIs) for distance and travel time, stratified by self-reported rural vs. urban residence

a) Distance to hospital (per 50 km), by rural vs. urban

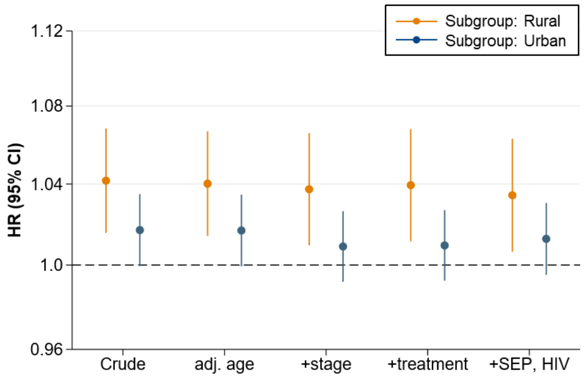

b) Travel time (per hour), by rural vs. urban

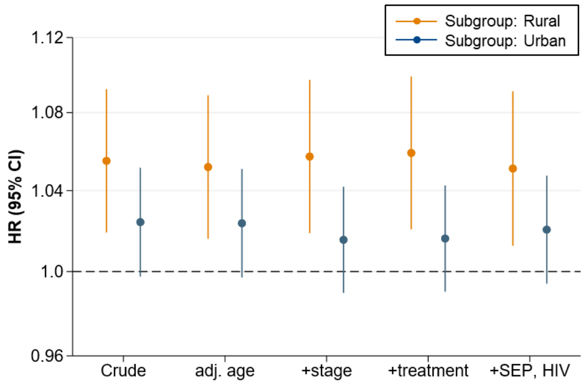

Table S3. Restricted mean survival time and 95% confidence intervals (CI) in rural vs. urban women diagnosed with breast cancer in the ABC-DO cohort study

	Mean survival time, years (95% CI)	Mean survival time, years (95% CI)		Rural – Urban difference in mean survival time	
		Rural (n=928)	*Urban (n=1173)*	*Years (95% CI)*	*Months (95% CI)*
Overall	2.60 (2.53, 2.67)	2.41 (2.30, 2.52)	2.76 (2.67, 2.85)	**-0.35 (-0.48, -0.21)**	**-4.1 (-5.8, -2.5)**
Study site/population					
Namibia, non-black	3.49 (3.27, 3.72)	2.70 (1.53, 3.86)	3.67 (3.50, 3.84)	-0.97 (-2.15, 0.21)	-11.6 (-25.8, 2.5)
Namibia, black	2.81 (2.65, 2.98)	2.58 (2.32, 2.83)	3.03 (2.85, 3.20)	**-0.45 (-0.76, -0.14)**	**-5.4 (-9.1, -1.7)**
Uganda	2.41 (2.25, 2.57)	2.29 (2.11, 2.47)	2.81 (2.55, 3.07)	**-0.52 (-0.84, -0.21)**	**-6.2 (-10.0, -2.5)**
Nigeria	2.10 (1.94, 2.26)	2.10 (1.83, 2.37)	2.10 (1.90, 2.29)	-0.00 (-0.33, 0.33)	-0.0 (-4.0, 4.0)
South Africa	2.83 (2.72, 2.95)	2.75 (2.57, 2.93)	2.89 (2.75, 3.02)	-0.13 (-0.36, 0.09)	-1.6 (-4.3, 1.1)
Zambia	2.43 (2.20, 2.66)	2.05 (1.65, 2.45)	2.70 (2.44, 2.97)	**-0.65 (-1.14, -0.17)**	**-7.9 (-13.7, -2.1)**
Distance to hospital					
≤50 km	2.63 (2.54, 2.71)	2.52 (2.37, 2.66)	2.68 (2.58, 2.79)	-0.17 (-0.35, 0.01)	-2.0 (-4.2, 0.2)
>50 km	2.56 (2.43, 2.68)	2.30 (2.15, 2.45)	2.93 (2.78, 3.08)	**-0.63 (-0.83, -0.42)**	**-7.5 (-10.0, -5.0)**
Travel time to hospital					
≤1 hr	2.62 (2.53, 2.70)	2.58 (2.41, 2.74)	2.70 (2.60, 2.81)	-0.13 (-0.32, 0.07)	-1.5 (-3.8, 0.8)
>1 hr	2.55 (2.41, 2.69)	2.30 (2.17, 2.44)	2.87 (2.71, 3.03)	**-0.57 (-0.78, -0.36)**	**-6.8 (-9.3, -4.3)**

Note: Restricted mean survival time and 95% CIs estimated using restricted mean survival time models truncated at 4.0 years. Abbreviations: CI, confidence interval.

Table S4. Time-dependent HRs for self-reported rural vs. urban residence, overall and among women living ≥50km from the hospital

Rural vs. urban	Adjustment	Peak HR (95% CI)[a]	Time at peak HR, years	Time period of elevated HRs, years	HR (95% CI) 0.5 years	HR (95% CI) 1.0 years	HR (95% CI) 2.0 years	HR (95% CI) 3.0 years
Overall	Crude	**1.44 (1.22-1.71)**	0.81	0.17 to 2.12	**1.41 (1.16-1.73)**	**1.45 (1.20-1.74)**	**1.21 (1.03-1.41)**	0.95 (0.76-1.19)
	Adj. age	**1.45 (1.22-1.72)**	0.81	0.17 to 2.13	**1.42 (1.16-1.73)**	**1.45 (1.20-1.75)**	**1.21 (1.03-1.42)**	0.95 (0.76-1.20)
	+stage	**1.41 (1.19-1.67)**	0.89	0.28 to 1.86	**1.36 (1.11-1.66)**	**1.41 (1.18-1.68)**	1.13 (0.96-1.33)	0.83 (0.66-1.03)
	+treatment	**1.41 (1.19-1.68)**	0.90	0.31 to 1.90	**1.35 (1.10-1.65)**	**1.41 (1.18-1.69)**	1.14 (0.97-1.34)	0.83 (0.66-1.04)
	+SEP, HIV	**1.30 (1.09-1.54)**	0.89	0.46 to 1.48	**1.25 (1.02-1.54)**	**1.30 (1.08-1.56)**	1.04 (0.88-1.23)	0.76 (0.61-0.96)
Subgroup: ≥50km	Crude	**1.93 (1.43-2.62)**	0.83	0.11 to 2.40	**1.92 (1.31-2.82)**	**1.93 (1.39-2.66)**	**1.50 (1.14-1.99)**	1.05 (0.75-1.47)
	Adj. age	**1.91 (1.40-2.59)**	0.83	0.13 to 2.38	**1.89 (1.29-2.77)**	**1.90 (1.37-2.64)**	**1.49 (1.13-1.98)**	1.05 (0.75-1.48)
	+stage	**1.77 (1.30-2.42)**	0.97	0.33 to 2.31	**1.65 (1.12-2.43)**	**1.78 (1.30-2.43)**	**1.47 (1.10-1.95)**	1.02 (0.73-1.42)
	+treatment	**1.78 (1.31-2.43)**	0.98	0.33 to 2.31	**1.65 (1.12-2.43)**	**1.79 (1.31-2.44)**	**1.47 (1.11-1.97)**	1.02 (0.73-1.42)
	+SEP, HIV	**1.75 (1.25-2.44)**	0.99	0.38 to 2.23	**1.61 (1.07-2.42)**	**1.75 (1.25-2.44)**	**1.45 (1.07-1.98)**	1.01 (0.71-1.43)

Note: HRs and 95% CIs estimated using flexible parametric survival models. Abbreviations: CI, confidence interval; HR, hazard ratio; SEP, socioeconomic position score. [a]Peak HR was defined as the highest lower CI.

Figure S7. Time-dependent HRs for self-reported rural vs. urban residence, overall and among women living ≥50km from the hospital

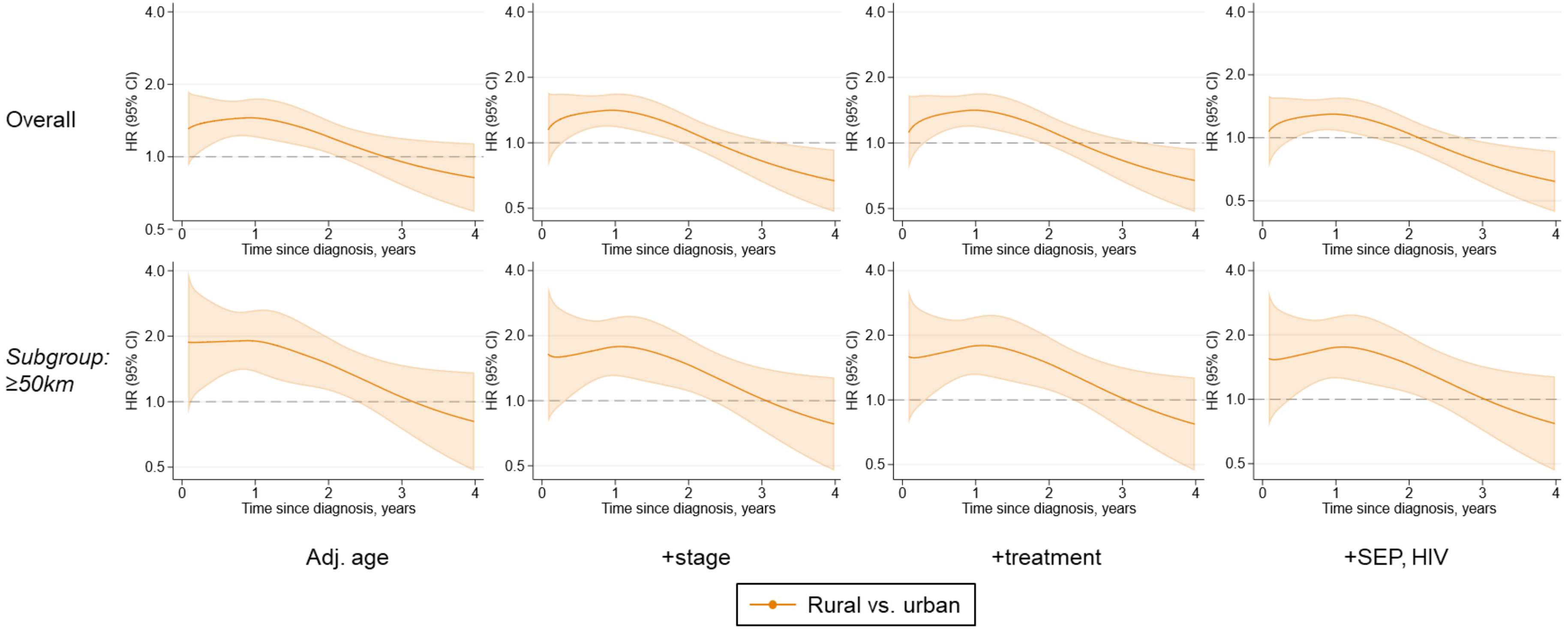

IV. SENSITIVITY ANALYSIS: GIS-BASED RURAL VS. URBAN

The main analyses regarding rural vs. urban disparities in survival are based on a dichotomization of the following four options in the baseline questionnaire regarding the level of urbanization of their area of residence: city or town (urban) and village or rural (rural).

As a sensitivity analysis, we used GIS methods to classify residential neighbourhood into rural vs. urban.

Methods

To do this, we constructed a Global Human Settlement Model (GHS-SMOD- R2023A) by applying the Degree of Urbanisation Stage I methodology recommended by UN Statistical Commission.[1] It was generated by integrating built-up surface data (GHS-BUILT-S R2023) and population data (GHS-POP R2023); these data are available from the Joint Research Centre Data Catalogue every five years, therefore the year 2015 was selected, in order to overlap with the ABC-DO baseline data collection period.[2] The resulting gridded surface had 1 km spatial resolution and contained seven classes which describe a continuum of urbanicity, which we assigned the following ordered values to allow logical arithmetic on the raster:[3] very low density rural grid cell (value=1), low density rural grid cell (2), rural cluster grid cell (3), suburban or peri-urban grid cell (4), semi-dense urban cluster grid cell (5), dense urban cluster grid cell (6), and urban centre grid cell (7). We then created buffers of 3 km to approximate the neighbourhood around each household location and overlaid them on the gridded surface. The mean value for each buffer was then extracted. Addresses with a mean urbanicity of ≥4 (the value corresponding to “suburban or peri-urban”) were classified as urban while the rest (<4) were classified as rural.

Results

Overall, there was broad agreement between the two methods. A high proportion of self-reported rural areas were classified as rural using GIS-methods, and the proportions decreased with increasing urbanicity of the self-reported categories (town, city; **Figure S8**). The HRs, although attenuated in magnitude compared to those for self-reported rural vs. urban residence, show a similar pattern as the original analysis.

Discussion

Self-reported rural vs. urban residence may be more valid in the SSA context than GIS-based globally standardized definitions of urbanicity (used in a sensitivity analysis here), which have led to surprising results in Africa; for example, identifying 67 cities vs. 20 formally recognized in South Africa.[4,5] Defined only by population size and density, they may not be suited to characterize the urban-rural continuum in SSA,[4,6] and misclassification could occur if, due to lack of formal addresses, some ABC-DO women reported recognizable landmarks in the nearest village. These would produce relatively small errors when calculating distance or travel time, but would misclassify rural women into more populated/urban areas.

Figure S8. Proportion of self-reported residential areas classified as rural (mean GIS urbanicity score <4) using GIS-based methods

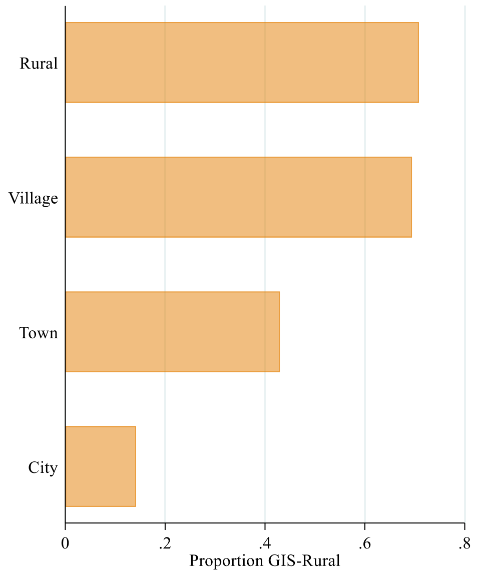

Table S5. Hazard ratios (HR) and 95% confidence intervals (CI) for all-cause mortality associated with geospatial characteristics, using the GIS-based rural vs. urban residence, in women diagnosed with breast cancer in the ABC-DO cohort study

Variable and Model	HR (95% CI)	Stratified HR (95% CI)	Stratified HRs (95% CI)	p_{int}
Rural vs. urban	*Overall (n=2101)*	*Distance >50 km (n=815)*	*Distance ≤50 km (n=1286)*	
Crude	**1.15 (1.00-1.32)**	1.23 (0.96-1.57)	1.00 (0.80-1.25)	0.22
+Age	1.14 (0.99-1.30)	1.19 (0.93-1.52)	1.00 (0.80-1.25)	0.30
+Stage	**1.17 (1.01-1.35)**	1.21 (0.94-1.56)	1.11 (0.88-1.39)	0.60
+Treatment	**1.18 (1.02-1.36)**	1.20 (0.93-1.55)	1.11 (0.89-1.40)	0.65
+SEP, HIV	1.13 (0.97-1.31)	1.18 (0.91-1.52)	1.08 (0.86-1.36)	0.64
Rural vs. urban	*Overall (n=2101)*	*Travel time >1 hr (n=734)*	*Travel time ≤1 hr (n=1367)*	
Crude	**1.15 (1.00-1.32)**	1.28 (0.97-1.69)	1.03 (0.84-1.26)	0.20
+Age	1.14 (0.99-1.30)	1.24 (0.94-1.64)	1.02 (0.83-1.25)	0.24
+Stage	**1.17 (1.01-1.35)**	**1.36 (1.03-1.82)**	1.09 (0.89-1.35)	0.22
+Treatment	**1.18 (1.02-1.36)**	**1.35 (1.01-1.79)**	1.10 (0.89-1.35)	0.25
+SEP, HIV	1.13 (0.97-1.31)	1.31 (0.98-1.74)	1.07 (0.87-1.32)	0.27
Distance (per 50 km)	*Overall (n=2101)*	*Rural (n=928)*	*Urban (n=1173)*	
Crude	**1.02 (1.01-1.04)**	**1.03 (1.01-1.04)**	1.00 (0.97-1.04)	0.20
+Age	**1.02 (1.01-1.04)**	**1.03 (1.01-1.04)**	1.00 (0.97-1.04)	0.26
+Stage	**1.01 (1.00-1.03)**	**1.02 (1.00-1.04)**	0.98 (0.95-1.02)	**0.08**
+Treatment	**1.02 (1.00-1.03)**	**1.02 (1.00-1.04)**	0.99 (0.95-1.02)	0.10
+SEP, HIV	**1.02 (1.00-1.03)**	**1.02 (1.00-1.04)**	0.99 (0.95-1.02)	**0.07**
Travel time (per 1 hr)	*Overall (n=2101)*	*Rural (n=928)*	*Urban (n=1173)*	
Crude	**1.04 (1.01-1.06)**	**1.04 (1.01-1.07)**	1.01 (0.96-1.06)	0.25
+Age	**1.04 (1.01-1.06)**	**1.04 (1.01-1.06)**	1.01 (0.96-1.06)	0.33
+Stage	**1.03 (1.00-1.05)**	**1.03 (1.01-1.06)**	0.98 (0.93-1.04)	**0.09**
+Treatment	**1.03 (1.00-1.05)**	**1.03 (1.01-1.06)**	0.99 (0.94-1.04)	0.11
+SEP, HIV	**1.03 (1.00-1.05)**	**1.04 (1.01-1.06)**	0.98 (0.93-1.04)	**0.08**

Note: HRs and 95% CIs estimated using Cox proportional hazards models stratified on study site/population (Namibia non-Black, Namibia Black, Uganda, Nigeria, South Africa, Zambia). Stratified HRs were produced from models with interaction terms between the main geospatial variable of interest and the effect modifier. Abbreviations: HR, hazard ratio; SEP, socioeconomic position score.

Section IV References

1. European Commission, and Statistical Office of the European Union, 2021. Applying the Degree of Urbanisation — A methodological manual to define cities, towns and rural areas for international comparisons — 2021 edition Publications Office of the European Union, 2021; ISBN 978-92-76-20306-3. 10.2785/706535
2. Schiavina M., Melchiorri M., Pesaresi M. (2023): GHS-SMOD R2023A - GHS settlement layers, application of the Degree of Urbanisation methodology (stage I) to GHS-POP R2023A and GHS-BUILT-S R2023A, multitemporal (1975-2030)European Commission, Joint Research Centre (JRC). doi:10.2905/A0DF7A6F-49DE-46EA-9BDE-563437A6E2BA
3. Macharia PM, Pinchoff J, Taylor C, et al. Exploring the urban gradient in population health: insights from satellite-derived urbanicity classes across multiple countries and years in sub-Saharan Africa. BMJ Global Health 2023;8:e013471.
4. Global Urban Observatory. Assessing the feasibility of applying a global definition of cities, urban and rural areas in support of global monitoring of SDGs and NUA urban targets. Lusaka, Zambia: United Nations Human Settlement Programme, European Commission, 2020.
5. UN-Habitat. A new city classification method shows Southern African countries more urbanized than thought. 2019. https://unhabitat.org/a-new-city-classification-method-shows-southern-african-countries-more-urbanized-than-thought (accessed January 12, 2024.
6. OECD, Commission TE, Food, et al. Applying the Degree of Urbanisation; 2021
