# Supplementary material for: Geospatial disparities in survival of patients with breast cancer in sub-Saharan Africa from the African Breast Cancer-Disparities in Outcomes cohort (ABC-DO): a prospective cohort study
Source: Lancet Glob Health. 2024 May 21;12(7):e1111–9. doi: 10.1016/S2214-109X(24)00138-4 (PMC11168938; doi:10.1016/S2214-109X(24)00138-4)
Supplement: Equitable Partnership Declaration [file mmc2.pdf]

THE LANCET
Global Health

Supplementary appendix 2

This Equitable Partnership Declaration (EPD) was submitted by the authors, and we reproduce it as supplied. It has not been peer reviewed. *The Lancet*'s editorial processes have not been applied to the EPD.

Supplement to: Supplement to: Kim J, Macharia PM, McCormack V, et al. Geospatial disparities in survival of patients with breast cancer in sub-Saharan Africa from the African Breast Cancer-Disparities in Outcomes cohort (ABC-DO): a prospective cohort study. *Lancet Glob Health* 2024; published online May 21. https://doi.org/10.1016/S2214-109X(24)00138-4.

Equitable Partnership Declaration questions

This Equitable Partnership Declaration is a statement being published online alongside papers at *The Lancet Global Health*, as a separate appendix, to allow researchers to describe how their work engages with researchers, communities, and environments in the countries of study. This is part of our broader goal to decolonise global health, handing control and leadership of research to academics and clinicians who are based in the regions of study, and to affected communities.

Please answer all questions with as much detail as possible, noting that all included information will be published open-access and it will be freely available online to all who wish to read it. If a question does not apply to your study, please state "Not applicable".

The format of and questions in this statement are currently in a pilot phase. Please email Dr Liam Messin (Liam.Messin@lancet.com; deputy editor) and Dr Kate McIntosh (Kate.McIntosh@lancet.com; senior editor) with any feedback, particularly if you find any questions unclear.

Researcher considerations

1. Please detail the involvement that researchers who are based in the region(s) of study had during a) study design; b) clinical study processes, such as processing blood samples, prescribing medication, or patient recruitment; c) data interpretation; and d) manuscript preparation, commenting on all aspects. If they were not involved in any of these aspects, please explain why.

This question is intended for international partnerships; if all your authors are based in the area of study, this question is not applicable.

This should include a thorough description of their leadership role(s) in the study. Are local researchers named in the author list or the acknowledgements, or are they not mentioned at all (and, if not, why)? Please also describe the involvement of early career researchers based in the location of the study. Some of this information might be repeated from the Contributors section in the manuscript. Note: we adhere to ICMJE authorship criteria when deciding who should be named on a paper.

**a) Study design:**<br>All country specific PIs were involved through the entire process of study design, study implementation and annual meetings. For this cohort, PIs met in person on an annual basis, the first time in April 2014 in Lyon and the last time in November 2022 in Uganda. All PIs (from 5 African countries) were present at every meeting.
**b) Clinical study processes:**<br>The country PIs supervised the clinical teams to extract all medical data. Statistical analyses were conducted at IARC, and all queries were discussed with the country specific PIs and teams on the ground.
**c) Data interpretation:**<br>All analyses were discussed with all country teams either in in-person meetings or during online meetings. The present work brought in GIS expertise not held in the core team, through a Kenyan expert.

**d) Manuscript preparation:**
The first draft of the manuscript was led by IARC postdoctoral fellow Dr Kim. All regional teams contributed to the revision of the manuscript and in particular, potential solutions for the local context.

2. Were the data used in your study collected by authors named on the paper, or have they been extracted from a source such as a national survey? ie, is this a secondary analysis of data that were not collected by the authors of this paper. If the authors of this paper were not involved in data collection, how were data interpreted with sufficient contextual knowledge?

   The Lancet Global Health *believe contextual understanding is crucial for informed data analysis and interpretation.*

The data used in this study were collected by the study team. The only generated data were GIS-based estimated travel distances, which were led by Dr Peter Macharia, thus he is the only additional senior author not part of the inception team who led the ABC-DO cohort study.

3. How was funding used to remunerate and enhance the skills of researchers and institutions based in the area(s) of study? And how was funding used to improve research infrastructure in the area of study?

   Potentially effective investments into long-term skills and opportunities within institutions could include training or mentorship in analytical techniques and manuscript writing, opportunities to lead all or specific aspects of the study, financial remuneration rather than requiring volunteers, and other professional development and educational opportunities.

   Improvements to research infrastructure could be funding of extended trial designs (such as platform trials) and use of master protocols to enable these designs, establishment of long-term contracts for research staff, building research facilities, and local control of funding allocation.

**Skills:** ABC-DO was intentionally observational, thus clinical interventions were limited. We did however provide a pathology course. We are co-supervising one PhD and with the South African team, which has a large group of young scientists, we continually provide analytical and study design advice for manuscripts.
**Research infrastructure:** ABC-DO was conducted with capacity building in mind. Two of the research nurses who conducted interviews in ABC-DO are now doing PhDs. One of these PhD topics is on the follow-up of the children of ABC-DO participants, i.e. in an examination of maternal orphans due to cancer. Several of the study interviewers were also invited to IARC (Lyon) to attend a two-week course in Cancer Epidemiology.<br><br>The study questionnaires have been shared and adapted for other studies now ongoing in Ethiopia and Eastern Europe.<br><br>For each country, a collaborative research agreement was set up between IARC and the respective grants office of the local institution (university or cancer association), who then dispersed the funds locally as agreed upon and with their indirect costs compensated.

4. How did you safeguard the researchers who implemented the study?
Please describe how you guaranteed safe working conditions for study staff, including provision of appropriate personal protective equipment, protection from violence, and prevention of overworking.

All researchers were employed by local institutions which have fair working policies in place and provided protection for study staff during the Covid 19 pandemic. In addition, all researchers were able to communicate directly with the international study PIs in case of late salaries and any problems could then be appropriately resolved if necessary and if appropriate. Local institutions had responsibility for hiring of local staff. Problems with late salaries occurred on rare occasions.

Benefits to the communities and regions of study

5. How does the study address the research and policy priorities of its location?

How were the local priorities determined and then used to inform the research question? Who decided which priorities to take forward? Which elements of the study address those priorities?

Breast cancer is a major cause of cancer death in all African countries, thus is a priority for the region. In terms of which factors to study in relation to its survival, these were jointly discussed at annual in person study team meetings which were attended by all country specific PIs, when new ideas were added and questions tailored to individual contexts.

6. How will research products be shared in the community of study?

For instance, will you be providing written or oral layperson summaries for non-academic information sharing? Will study data be made available to institutions in the region(s) of study? The Lancet Global Health *encourages authors to translate the summary (abstract) into relevant languages after paper editing; do you intend to translate your summary?*

We are in discussion with cancer associations and relevant ministries of health regarding the main findings, whose major implications are for health system referrals. We are also developing follow-on studies to improve survival using setting appropriate strategies, e.g. improving breast health awareness, shortening delay times to diagnosis and improving treatment compliance.

7. How were individuals, communities, and environments protected from harm?

a) How did you ensure that sensitive patient data was handled safely and respectfully? Was there any potential for stigma or discrimination against participants arising from any of the procedures or outcomes of the study?

Confidential data were held locally and an online-line platform gave international access only to anonymized data unless names and addresses were needed by individual researchers (e.g. for GPS coding).

b) Might any of the tests be experienced as invasive or culturally insensitive?

The study involved regular follow-up contact with the study participants including a QOL interview, some questions of which (e.g. on sexual health) might have been sensitive.

c) *How did you determine that work was sensitive to traditions, restrictions, and considerations of all cultural and religious groups in the study population?*

All questionnaires were first approved by each local ethics committee. Thereafter, they were pre-tested by the local team and sensitive questions were identified during role play situations. There were options for participants not to answer a question that they preferred not to.

d) *Were biowaste and radioactive waste disposed of in accordance with local laws?*

Not applicable.

e) *Were any structures built that would have impacted members of the community or the environment (such as handwashing facilities in a public space)? If so, how did you ensure that you had appropriate community buy-in?*

Not applicable. ABC-DO is a clinical observational study.

f) *How might the study have impacted existing health-care resources (such as staff workloads, use of equipment that is typically employed elsewhere, or reallocation of public funds)?*

ABC-DO is a clinical observational study. No equipment was used that would have been as per routine clinical practice. The interviewer's time was paid for by the study.

8. Finally, please provide the title (eg, Dr/Prof, Mr/Mrs/Ms/Mx), name, and email address of an author who can be contacted about this statement. This can be the corresponding author.

Name: Valerie McCormack
Email: mccormackv@iarc.who.int
